# Plasmonic Lightning‐Rod Effect

**DOI:** 10.1002/nap2.70348

**Published:** 2026-11-06

**Authors:** Vlastimil Křápek, Rostislav Řepa, Michael Foltýn, Tomáš Šikola, Michal Horák

**Affiliations:** ^1^ Brno University of Technology Central European Institute of Technology Brno Czechia; ^2^ Brno University of Technology Institute of Physical Engineering Brno Czechia

**Keywords:** electron energy loss spectroscopy, surface plasmon, electric field, radius of curvature, curvature, plasmon, physics, electromagnetic simulation

## Abstract

The plasmonic lightning‐rod effect refers to the formation of a strong electric near field of localized surface plasmons at the sharp features of plasmonic antennas. Although this effect is intuitively utilized in the design and optimization of plasmonic antennas, the relation between the magnitude of the electric field and the local curvature of the plasmonic antenna has not yet been rigorously established. Here, we provide such a study. We design sets of plasmonic antennas that allow us to isolate the role of the local curvature from other effects influencing the field. The electric near field is inspected by electron energy‐loss spectroscopy and electrodynamic simulations. We confirm the existence of the plasmonic lightning‐rod effect, demonstrate its decoupling from concurrent effects influencing the field, and establish its quantitative description, showing that its strength can be interpreted as comparable to that of the electrostatic lightning‐rod effect. We also provide a simple empirical formula for the spatial dependence of the field, which includes the effective radius of curvature significantly smaller than the geometrical radius.

## Graphical abstract

How strongly does local curvature control plasmonic field enhancement? By isolating curvature effects in carefully designed plasmonic antennas, the plasmonic lightning‐rod effect is quantitatively established. An effective radius of curvature, smaller than the geometric radius, emerges from the induced charge distribution, providing a new framework for understanding near‐field enhancement.

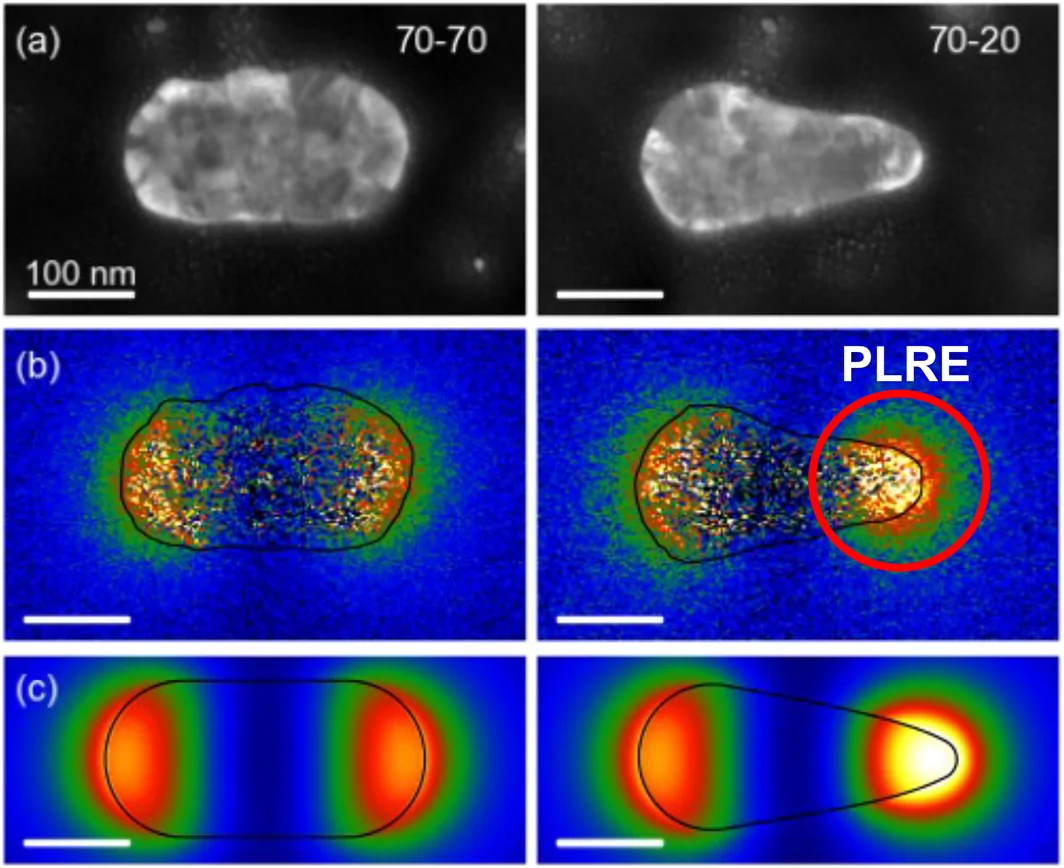

## 1 Introduction

Plasmonic antennas [1] (PAs) represent a class of open photonic cavities formed by metallic nanostructures that support localized surface plasmons (LSP). The excitation of LSP is related to the formation of a spatially confined and enhanced electromagnetic field, which gives rise to enhanced light–matter interaction. This effect is utilized in a wide range of applications, including plasmon‐enhanced spectroscopy (photoluminescence spectroscopy [2, 3], Raman spectroscopy [4, 5], and electron paramagnetic resonance spectroscopy [6]), energy harvesting [7], strong light–matter coupling [8, 9], sensing [10, 11], and medicine [12, 13].

The design and optimization of PAs exploit ingenious concepts, including transformation optics [14, 15], plasmonic circuit models [16, 17, 18, 19], Babinet's principle [20, 21, 22, 23], hybridization models [24, 25], dimension scaling based on the effective dispersion relation [26], and material aspects [27]. One of the widely utilized concepts is the so‐called plasmonic lightning‐rod effect (PLRE), which refers to the local enhancement of the electric field at the parts of PAs with high local curvature.

Applications of PLRE include single‐molecule fluorescence [2], plasmon–exciton strong coupling [9], optical spectroscopy [28, 29], energy‐storing and energy‐generating devices [30], energy harvesting [7, 31], photocatalysis [32], multichannel photodiode detectors [33], multibeam photoemission cathodes [34], single‐photon emission for quantum communication [35], sensing represented, for example, by biosensors for nitrite detection [36], local sensing by lens‐free tip‐enhanced Raman spectroscopy with 1 nm spatial resolution [37], and a single atomic protrusion forming a picocavity that allows the study of single‐molecule optomechanics [38].

Although widely used, PLRE is not yet fully understood, despite studies addressing its particular aspects [39, 40, 41, 42, 43, 44], as detailed in the Supporting Information S1. Specifically, a quantification of PLRE isolated from concurrent effects influencing the electric field has not yet been reported. Here, quantification is understood as finding a quantitative relation between the magnitude of the induced electric field and the local curvature of PAs. To establish such a relation, it is essential to vary the local radius of curvature of PAs, typically by studying multiple PAs. However, the change in the local radius can affect additional parameters of PAs and the LSP hosted by them, such as the resonance energy or radiative losses. In other words, PLRE is often expressed simultaneously with concurrent effects influencing the magnitude of the induced electric field, and quantification of PLRE requires its decoupling or isolation from the concurrent effects.

Our study fills this gap by addressing and establishing the quantitative relation between the local electric field of LSP and the local radius of curvature of PAs. We identify and control additional possible effects contributing to the field magnitude in order to isolate the pure effect of the curvature.

## 2 Design

The lightning‐rod effect was originally formulated in electrostatics, where it can be demonstrated and quantified for a system of two distant metallic spheres [Figure 1a]. For a single homogeneous metallic sphere of radius R charged with a charge Q, the electrostatic potential and electric field on its surface are given by $\varphi =k_{e}Q/R$ and $E=k_{e}Q/R^{2}$, respectively, where $k_{e}=1/4\pi \varepsilon_{0}$ is the Coulomb constant. We will now consider two such spheres (labeled with Subscripts 1 and 2) with radii $R_{1}$ and $R_{2}$. The spheres are far enough apart for the mutual electrostatic interaction to be negligible and are conductively connected with a long thin wire. When the system is charged, the total charge Q redistributes so that the electrostatic potentials of both spheres are equal, $\varphi_{1}=\varphi_{2}=\varphi$, yielding $E_{i}=\varphi /R_{i}$. The field at the surface of the spheres is inversely proportional to their local radius. For conductors of more complex shapes, this simple quantitative dependence does not hold, but the qualitative trend of larger fields for smaller local radii is preserved.

**FIGURE 1:**
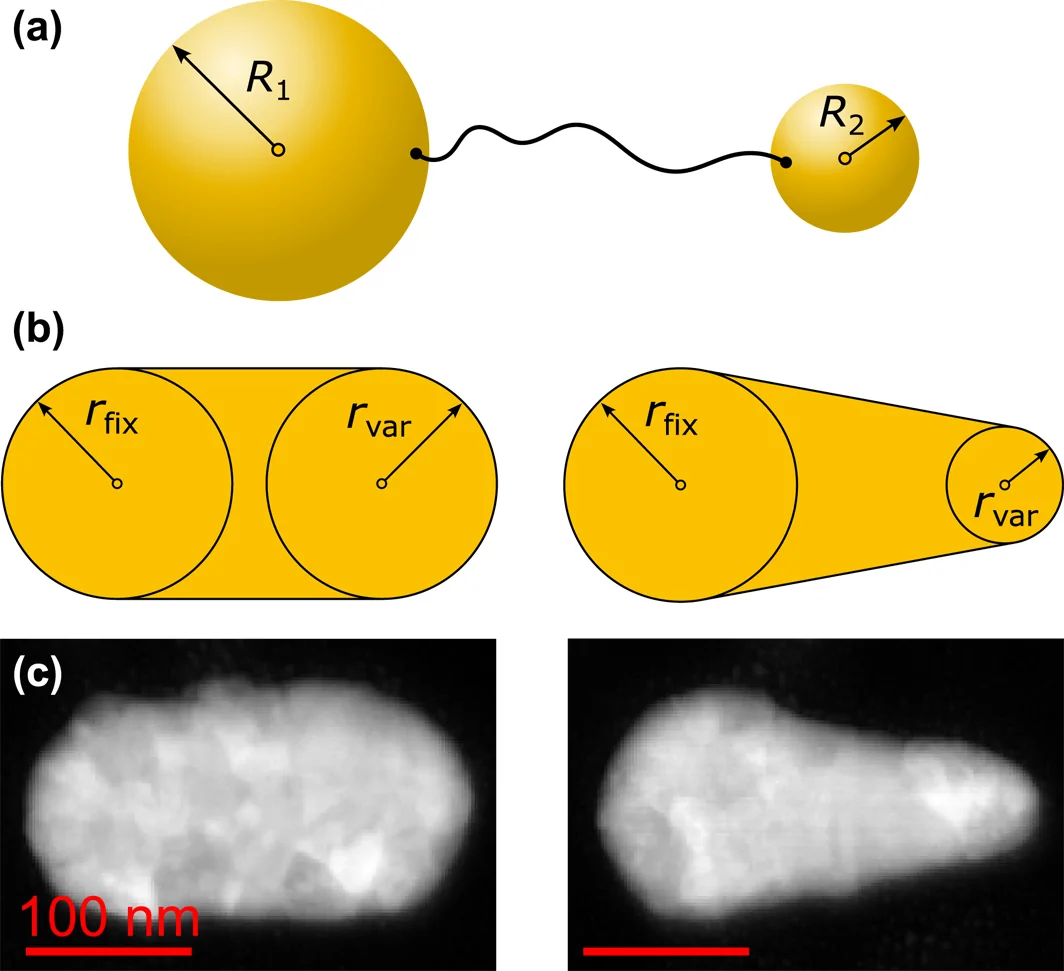
(a) A scheme of the setup demonstrating the electrostatic lightning‐rod effect. (b) Design of the plasmonic beetroot—a platform for studying PLRE. (c) HAADF STEM images of two fabricated PBs with $r_{\text{fix}}=70\;nm$ and $r_{\text{var}}=70\;nm$ (left) and 20 nm (right).

PLRE differs in two important aspects. First, both propagating and localized surface plasmon polaritons (SPPs) are evanescent waves with naturally confined electric fields, even for zero curvature of the supporting PA. As an example, we will consider a planar metal–vacuum interface. When such an interface is statically charged with a surface charge density σ, it produces a homogeneous field in the vacuum with the magnitude $E_{\text{stat}}=\sigma /\varepsilon_{0}$. On the other hand, when an SPP is excited at such an interface, the related electric field decays exponentially into the vacuum as $E_{\text{SPP}}\left(z\right)=E_{0}\;\exp\left(-\kappa z\right)$ [45], where z denotes the distance from the interface into the vacuum and $\kappa =\left(\omega /c\right)\;\cdot \;{\left[1/\left|\varepsilon_{m}+1\right|\right]}^{1/2}$ is the decay constant (with ω, c, and $\varepsilon_{m}$ representing the angular frequency of the SPP, the speed of light in vacuum, and the dielectric constant of the metal, respectively). Second, in electrostatics, the metallic object is charged, whereas in plasmonics, the PA is quasineutral with charge oscillations related to LSP driven by an external excitation. Distinct PAs respond differently to the same excitation, yielding different magnitudes of the induced charge and different magnitudes of the induced electric near field. In the following, we denote this as the charge reservoir effect.

The electric field of PAs is affected by all the above‐mentioned effects (PLRE, evanescent confinement, and charge reservoir), and possibly other effects not yet identified. To isolate the PLRE, we need to either remove or account for the variations of any other effects when changing the local curvature of PAs. To this end, we design a set of planar rod‐shaped PAs with a fixed length and two cylindrical terminations [Figure 1b]. In the following, we will denote these PAs as plasmonic beetroots (PBs). In each set of plasmonic beetroots, one of the terminations has a fixed radius $r_{\text{fix}}$ and serves as a reference, whereas the other termination has a variable radius $r_{\text{var}}$ and is utilized to monitor the PLRE. The fixed length of all PBs in each set shall ensure similar energies of LSP modes and thus exclude variations in evanescent confinement. Variations in any effects contributing to the electric field other than the PLRE shall be reflected in variations of the reference field at the fixed‐radius termination. The electric field recorded at the variable‐radius termination (labeled as the test field, $E_{\text{test}}$) and normalized by the reference field at the fixed‐radius termination, $E_{\text{ref}}$, shall then reflect solely the effect of the local curvature. The dependence of the normalized test field (labeled in the following as the enhancement factor, $EF=E_{\text{test}}/E_{\text{ref}}$) on the local radius of curvature $\left(r_{\text{var}}\right)$ represents the desired quantification of the PLRE isolated from the concurrent effects influencing the electric field.

## 3 Methods

Our methodology combines electromagnetic simulations with experimental electron energy‐loss spectroscopy (EELS). The simulations are used for a full quantitative vectorial characterization of the electric field, whereas EELS is utilized to verify the simulations and also provides qualitative insight into the field [25, 46, 47, 48].

For the fabrication of PBs, a thin gold film with a thickness of 30 nm is deposited by ion‐beam‐assisted sputtering onto a standard 30‐nm‐thick silicon nitride membrane for electron microscopy (Agar Scientific), and the desired PB shape is achieved by nanofabrication with focused ion beam milling (Tescan Lyra 3, ${Ga}^{+}$ ions at 30 keV). The high‐angle annular dark‐field (HAADF) images of fabricated PBs obtained in a scanning transmission electron microscope (STEM) are shown in Figure 1c. Further structural and chemical properties of PAs fabricated with this procedure are reported in Refs. [49, 50].

EELS measurements were carried out in a STEM (FEI Titan with GIF Quantum spectrometer; primary beam energy, 120 keV; probe current, 100 pA; acquisition time, 0.5 ms). The full width at half maximum of the zero‐loss peak was found to range from 0.10 to 0.12 eV. The raw data were normalized to yield the loss probability, from which the zero‐loss peak and the background were subtracted to isolate the contribution of LSP. Individual peaks corresponding to LSP resonances were fitted with Lorentzian spectral profiles.

Theoretical modeling was performed using the software package MNPBEM [51, 52]. The geometry of the modeled PBs matched the design parameters of the fabricated structures. The dielectric function of gold was taken from Ref. [53], and the dielectric constant of the silicon nitride membrane was set equal to 4, which is a standard approximation in the considered spectral region.

## 4 Results

### 4.1 Loss Probability

We fabricated two series of PBs, the first with the fixed termination radius $r_{\text{fix}}=70\;nm$ and the variable radius $r_{\text{var}}$ varied from 70 to 20 nm in steps of 10 nm, and the second with $r_{\text{fix}}=50\;nm$ and $r_{\text{var}}$ varied from 50 to 20 nm in steps of 10 nm. The length of all PBs was fixed at L=300nm. The actual dimensions might differ slightly from these design dimensions. For each combination of $r_{\text{fix}}$ and $r_{\text{var}}$, ten individual PBs were fabricated, and the three that the best corresponded to the designed shape were selected for further analysis.

The loss probability is represented in the spectral domain in Figure 2 and in the spatial domain in Figure 3. The experimental loss probability spectra [Figure 2a,b] exhibit a pronounced LSP resonance at an energy of about 1.1 eV, identified as the longitudinal dipole (LD) resonance. This identification is based on the spatial distribution of the loss probability (Figure 3) and the calculated electric field (not shown), as explained in Ref. [25]. The LD resonance is spectrally well separated from the higher‐order resonances. By fitting Lorentzian profiles, we obtained the principal LD resonance parameters: the resonance energy and the Q factor (defined as the resonance energy divided by the full width at half maximum). These parameters are independent of the position of the electron beam within the experimental error, which proves that the loss probability in this spectral region is dominated by a single LSP resonance. In the following, we will focus on the LD resonance; that is, all quantities will be evaluated for the LD resonance or at its central energy.

**FIGURE 2:**
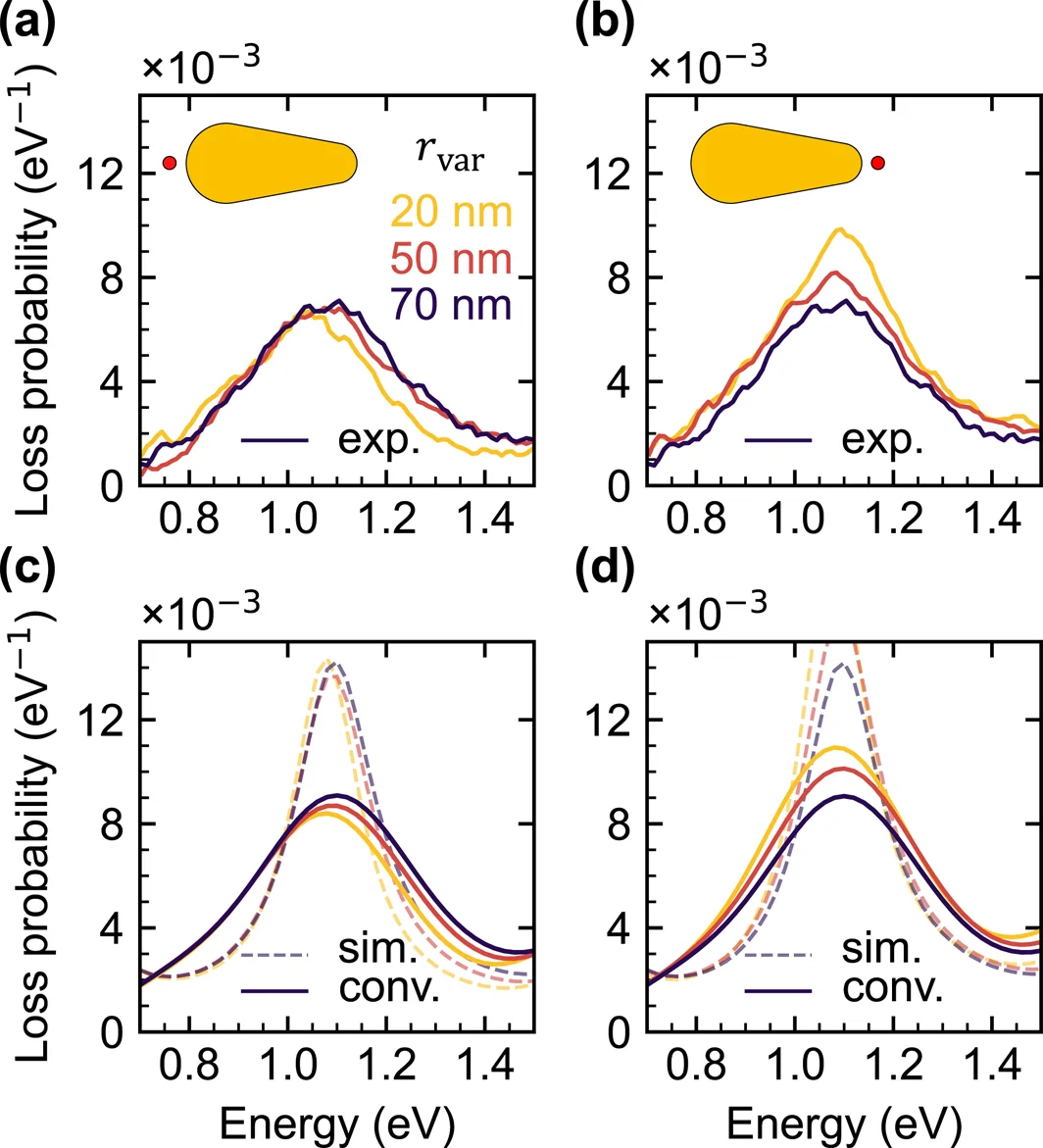
Loss probability spectra for PBs with $r_{\text{fix}}=70\;nm$ and three values of $r_{\text{var}}$. The electron beam positions are indicated in the insets [10 nm from the PB boundary at the fixed‐radius termination (left panels) or at the variable‐radius termination (right panels)]. (a, b) Experimental spectra. (c, d) Calculated spectra (solid lines) and calculated spectra convolved with a Gaussian (with a full width at half maximum of 0.27 eV) representing instrumental broadening.

**FIGURE 3:**
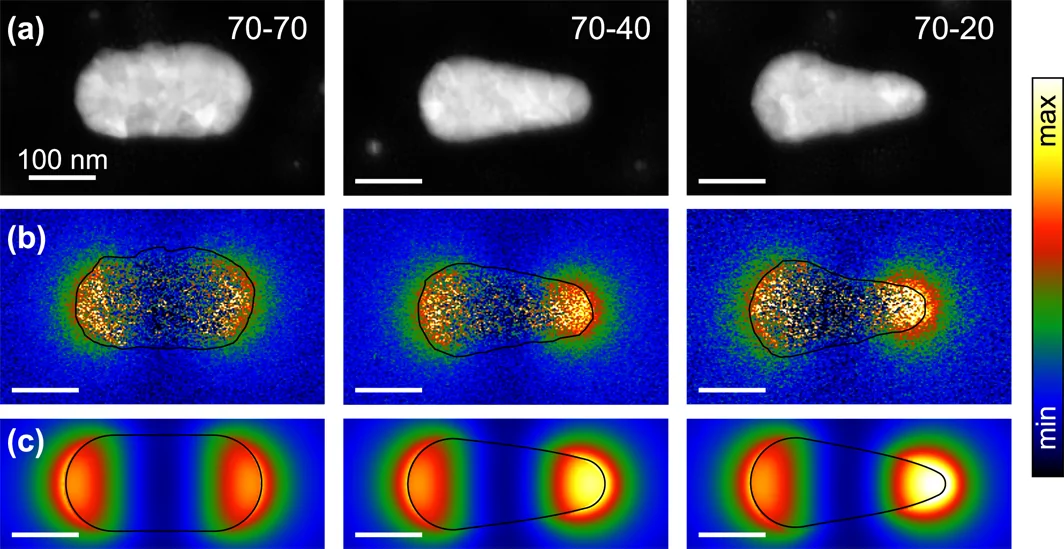
Loss probability maps for the same PBs as in Figure 2 at the energy of the LD resonance (approximately 1.1 eV). (a) HAADF STEM images of PBs. The numbers in the upper right corners represent $r_{\text{fix}}$−$r_{\text{var}}$ in nanometers. (b) Experimental maps averaged over the energy window with a width of 0.1 eV. (c) Calculated maps. Black contours in Panels (b) and (c) represent the boundaries of PBs.

Interestingly, the global parameters of the LD resonance (the resonance energy and the Q factor) are very similar for all PBs in both sets, with the energy in the interval 1.04–1.07 eV and the Q factors in the interval 2.8–3.5. In other words, changing $r_{\text{var}}$ might influence the local response of PBs but does not affect the global LSP resonance parameters. This finding underlines the suitability of the PB sets to isolate the PLRE.

In contrast to the global parameters of the LD resonance, the peak amplitude of the loss probability at the LD resonance energy varies with the position of the electron beam (as shown in Figures 2 and 3). The loss probability is closely related to the electric near field induced in PBs by the probe electron, as discussed in Refs. [25, 46, 47, 48]. The observation of the increased loss probability near the variable termination of the PB as $r_{\text{var}}$ is reduced [Figure 3b, left to right, right side of the PBs; Figure 2b] is an indirect manifestation of the PLRE.

Theoretical loss probability [Figures 2c,d and 3c] reproduces the experimental loss probability (Figures 2a,b and 3b) with full qualitative and good quantitative agreement (see the Supporting Information S1 for details). The experimental EELS therefore provides validation of our theoretical simulations, which provide additional characteristics unavailable in the experiment, in particular, a full vectorial electric field related to the LSP resonances.

### 4.2 Electric Field

Next, we will discuss the electric field (retrieved from a numerical solution of Maxwell's equations) induced by the probe electron due to the excitation of the LD resonance in the PB. We stress that the induced field does not include the excitation field of the probe electron. It is calculated as the difference between the total field and the field of the probe electron in vacuum. We examine the test field $E_{\text{test}}$ recorded at the variable‐radius termination (where the PLRE varies) and the reference field $E_{\text{ref}}$ recorded at the fixed‐radius termination (where the PLRE is constant). We evaluate the field at the long horizontal axis of the PB, in the middle of its height, and at a distance x from the PB boundary, as indicated in the inset in Figure 4a; the field value obtained right at the surface would suffer from a large numerical error.

**FIGURE 4:**
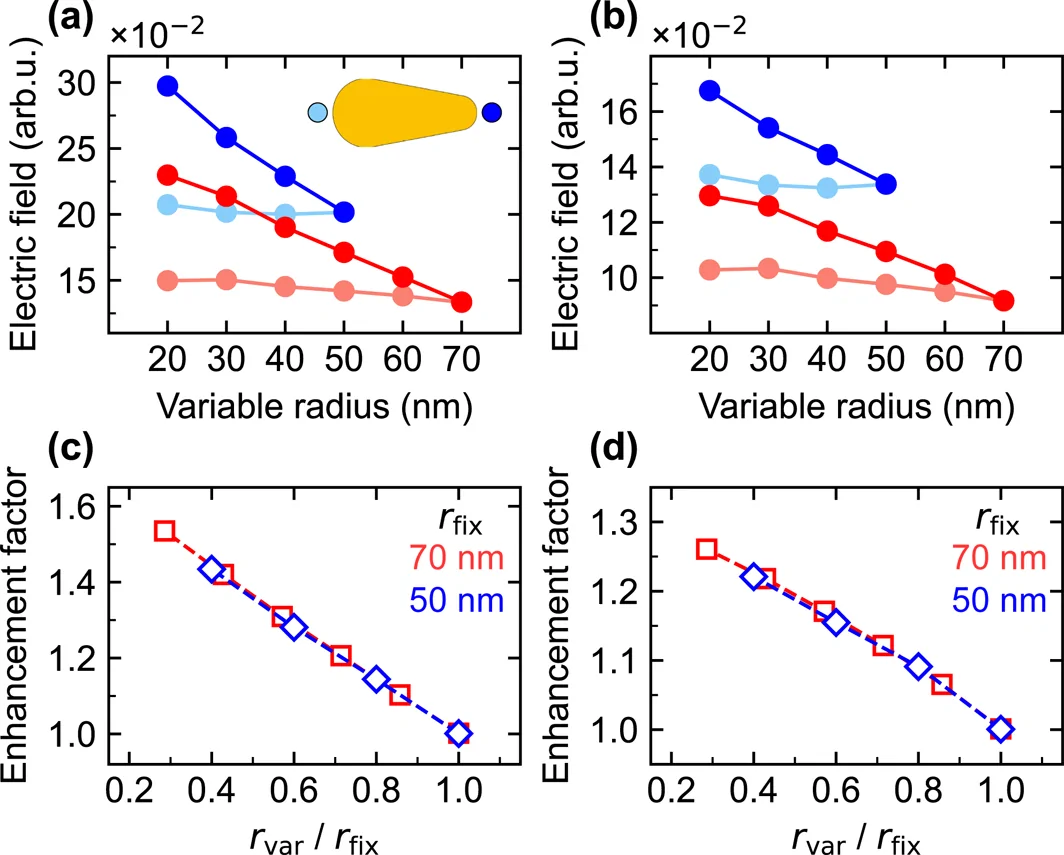
(a, b) Calculated test field $E_{\text{test}}$ (dark blue/red) and reference field $E_{\text{ref}}$ (light blue/red) as functions of the variable radius $r_{\text{var}}$ for fixed radii $r_{\text{fix}}$ of 50 nm (blue) and 70 nm (red) at distances from the PB, x, of 10 nm (a) and 20 nm (b). The inset indicates the positions where the field is evaluated. (c, d) Enhancement factors EF obtained for the fields from Panels (a) and (b), respectively.

In the calculations of the field, we set the electron beam so that it crosses the long axis of the PB 10 nm away from the termination *opposite* to the one where the field is recorded [that is, the beam is positioned at the variable‐radius termination (dark‐blue spot in the inset of Figure 4a) for the field $E_{\text{ref}}$ recorded at the fixed‐radius termination (light‐blue spot in the same inset) and vice versa]. By maintaining a large distance between the probe electron and the recorded field, we suppress the numerical instabilities of the simulations. Further discussion is provided in the Supporting Information S1.

The test and reference electric field magnitudes are plotted in Figure 4a,b as functions of the variable radius $r_{\text{var}}$ for two values of the distance x. We clearly observe that, as the radius decreases (i.e., the curvature increases), the test field increases. The reference field varies only weakly with $r_{\text{var}}$, but some variations are still present, indicating the influence of effects other than the PLRE. To remove them and isolate the pure effect of the local curvature, we have defined the enhancement factor as the ratio of the test and reference fields, $EF=E_{\text{test}}/E_{\text{ref}}$, and plotted it as a function of the relative radius $r_{\text{var}}/r_{\text{fix}}$ in Figure 4c,d. We observe an unequivocal increase in the enhancement factor with decreasing relative radius, a clear manifestation and quantification of the PLRE isolated from the concurrent effects influencing the electric field. It is noteworthy that the enhancement factor in both sets of PBs (i.e., with $r_{\text{fix}}$ values of 50 and 70 nm) is determined by the same function of $r_{\text{var}}/r_{\text{fix}}$. This resembles the case of the electrostatic lightning‐rod effect (ELRE) in the geometry of Figure 1a, where EF evaluated at the surfaces of the spheres is a universal function of $r_{\text{var}}/r_{\text{fix}}$ ($E_{\text{test}}\;\propto \;1/r_{\text{var}}$, $E_{\text{ref}}\;\propto \;1/r_{\text{fix}}$, $EF=r_{\text{fix}}/r_{\text{var}}$).

At first glance, the values of EF seem to be rather low. To illustrate this impression, we will inspect the case of $r_{\text{fix}}=70\;nm$, $r_{\text{var}}=20\;nm$, and x=20nm, where we find the PLRE enhancement factor EF=1.26. For the ELRE in the geometry of Figure 1a, we would obtain ${EF}_{\text{ELRE}}=\left(r_{\text{fix}}+x\right)/\left(r_{\text{var}}+x\right)=2.25$. A natural interpretation of this observation would be that the PLRE is weak compared to the ELRE. However, we propose a different interpretation. Using an empirical model (described below), the calculated electric field can be interpreted as originating from an effective charge (which is an empirical parameter of the model, not a real charge) located at a distance $r_{\text{eff}}$ (the so‐called effective radius of curvature) from the PB boundary. The effective radii of curvature related to the spatial distribution of the induced charge (and defined as empirical parameters obtained by fitting Equation (1) to the field profile shown in Figure 5; see the Supporting Information S1 for further details) are considerably smaller than the geometrical radii. In the case considered above, we obtain $r_{\text{fix}}^{\text{eff}}=16\;nm$ and $r_{\text{var}}^{\text{eff}}=6\;nm$, yielding the effective enhancement factor for the ELRE, ${EF}_{\text{ELRE}}^{\text{eff}}=\left(r_{\text{fix}}^{\text{eff}}+x\right)/\left(r_{\text{var}}^{\text{eff}}+x\right)=1.4$, comparable to the EF=1.26 for the PLRE.

**FIGURE 5:**
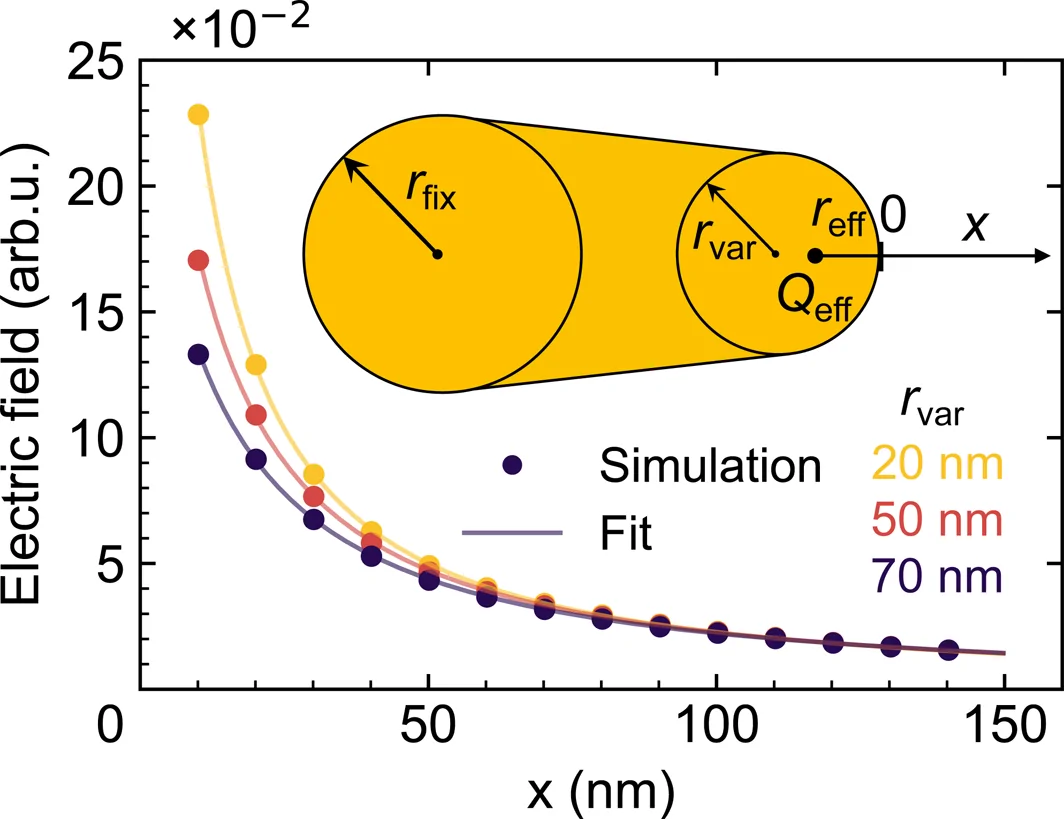
The induced electric field near the variable‐radius termination along the long axis of the PB (the line shown in the inset): the field obtained from electrodynamic simulations (symbols) and the fit by the empirical model (lines). The inset shows the parameters of the empirical model.

We will now discuss in more detail the empirical model for the induced field (restricting the analysis to the field at the long axis of the PB). We have tested several such models, and we present here the minimum model, surprisingly simple, which reliably reproduces the real field. It consists of a single effective charge $Q_{\text{eff}}$ induced inside the PB at a distance $r_{\text{eff}}$ from the PB boundary (the so‐called effective radius). We include the so‐called dimensionality factor d, which describes how fast the field decays into the dielectric, with values of 2 and 1 corresponding to ideal point and linear charges, respectively. The equation for the electric field E(x) in the vacuum at a distance x from the PB reads as follows:

(1) $$E\left(x\right)=\frac{Q_{\text{eff}}}{{\left(r_{\text{eff}}+x\right)}^{d}}.$$

The profile of the induced electric field near the variable‐radius termination (i.e., the test field) along the long axis of the PB is shown in Figure 5. The empirical model describes the field obtained from electrodynamic simulations very well. It is interesting to inspect the parameters of the model obtained from fitting the actual field (in the distance range from 10 to 150 nm). Importantly, the effective radius $r_{\text{eff}}$ is very small, between 6 and 16 nm, and increases as $r_{\text{var}}$ increases, ranging between $(0.2-0.3)\times r_{\text{var}}$. In other words, the effective charge is positioned far from the center of the cylindrical termination, near the edge of the PB. This is in striking contrast with the electrostatic case considered in Figure 1b, where the charge exhibits a spherically symmetric distribution around the center of the sphere. In PLRE, the geometrical radius of curvature of the PA significantly differs from the effective radius defined by the spatial distribution of the induced charge. Further, the dimensionality parameter d is within the range of 1.2±0.1, that is, corresponding rather to a linear effective charge. These facts explain rather quantitatively the strength of PLRE, as shown in Figure 4 and discussed above (see also the Supporting Information S1).

The low effective radius has important consequences for the design of PAs. For example, when the field is to be enhanced 10 nm from a certain protrusion of the PA, reducing the radius of the protrusion from 50 to 20 nm (expected effective radius reduction from 15 to 6 nm for $r_{\text{eff}}=0.3r$) is expected to increase the field by a fair factor of 25/16=1.6, but further reduction to 10 nm (expected effective radius of 3 nm) brings an expected reduction by a factor of 16/13=1.2, which might not be worth the fabrication effort.

The universality of our results is demonstrated by addressing additional instances discussed in the Supporting Information S1, including additional shapes of PAs, modified substrates, and additional excitation of PAs.

## 5 Conclusion

We have confirmed the existence of the plasmonic lightning‐rod effect, that is, the formation of an enhanced induced electric field related to a specific LSP resonance near highly curved parts of the plasmonic antenna. By employing sets of plasmonic beetroots that allow comparison of the test and reference fields, we isolated the effect of the local curvature from other effects influencing the induced electric field, in particular, evanescent confinement and the charge reservoir. The ratio of the electric fields recorded at two different parts of a single plasmonic beetroot (referred to as the enhancement factor EF) depends primarily on the ratio of the local radii (and the distance from the beetroot at which the fields are recorded), whereas its dependence on other parameters, such as the length and width of the beetroot, the substrate, or the means of excitation, is rather weak. This finding underlines the universality of the plasmonic lightning‐rod effect. Quantitative analysis demonstrated that the plasmonic lightning‐rod effect is weaker than the electrostatic lightning‐rod effect for the same ratio of geometrical radii. However, it can be interpreted as comparably strong using an empirical model in which the induced electric field is attributed to a single effective charge reflecting the true spatial distribution of the induced charge. The induced electric field exhibits a quasihyperbolic ($r^{-\left(1.2\pm 0.1\right)}$ with the substrate, $r^{-\left(1.7\pm 0.2\right)}$ without the substrate) decay with the distance from the effective charge. Importantly, the effective charge is located close to the boundary of plasmonic antennas, reducing their effective radius of curvature significantly in comparison to their geometrical radius.

The data that support the findings of this article are openly available [54].

## Author Contributions

**Vlastimil Křápek:** conceptualization, supervision, investigation, formal analysis, writing – original draft, writing – review and editing. **Rostislav Řepa:** investigation, formal analysis, writing – original draft, writing – review and editing. **Michael Foltýn:** investigation. **Tomáš Šikola:** funding acquisition. **Michal Horák:** investigation.

## Conflicts of Interest

The authors declare no conflicts of interest.

## Supporting information


Supporting Information S1

## Data Availability

The data that support the findings of this study are openly available in Zenodo at http://dx.doi.org/10.5281/zenodo.13988933.
