# Supplementary material for: Plasmonic Lightning‐Rod Effect
Source: Nanophotonics. 2026 Nov 6;15(21):e70348. doi: 10.1002/nap2.70348 (PMC13741146; doi:10.1002/nap2.70348)
Supplement: Supplementary file 1 [file NAP2-15-e70348-s001.pdf]

Plasmonic lightning-rod effect: Supplemental Material

Vlastimil Křápek,[1,2,*] Rostislav Řepa,[1,2] Michael Foltýn,[1,2] Tomáš Šikola,[1,2] and Michal Horák[1,†]

[1] *Brno University of Technology, Central European Institute of Technology, Purkyňova 123, 612 00 Brno, Czechia*
[2] *Brno University of Technology, Institute of Physical Engineering, Technická 2, 616 69 Brno, Czechia*

S1. LITERATURE OVERVIEW FOR PLASMONIC LIGHTNING-ROD EFFECT

The plasmonic lightning-rod effect (PLRE) is well known to the community of researchers in the field of plasmonics and is also widely exploited in applications. It is therefore rather surprising that an important substance of the PLRE has not yet been studied: a relation between the local electric field and local curvature, isolated from concurrent effects influencing the field. To verify this statement, we have performed an extensive search of the literature. The references to relevant works are provided in the main paper. The purpose of the text in this Supplemental Material is to summarize the achievements of the references so that our readers need not to survey them themselves. The available results can be categorized into two groups based on their relation to PLRE.

(i) *Quantitative relations between the radius of curvature and a response function*, possibly the local field. Such results are often scattered as the side results in the studies with a different principal focus.

García-Etxarri et al. [1] reported one of the first systematic studies of local curvature of plasmonic antennas (PAs). They theoretically compared three PAs with the fixed volume: two nanorods with hemispherical terminations and a sphere corresponding to a degenerate nanorod. When the radius of the termination was varied, the field increased as the curvature increased; the authors even used for the first time the term *lightning-rod effect* in relation to plasmonics. From our perspective, their finding is affected by the fact that fixing the volume does not prevent variation of other effects contributing to the field enhancement. Among others, the resonance energy significantly differs among the three compared PAs. The authors state that some effects contributing to the field enhancement are expressed simultaneously and cannot be isolated [1], and indeed, we are convinced that the role of the local curvature was not isolated from the other effects.

Another prominent study was reported by Shi et al. [2], who illuminated single bow-tie PA with a strong continuous laser light (the incident power density of 1 $\mathrm{TW/cm^2}$ increased locally in the bowtie gap up to 100 $\mathrm{TW/cm^2}$) and monitored third-harmonic generation (THG). The temporal evolution of the THG signal exhibited a brief (about 15 s) increase followed by a steady decay. The origin of this evolution was tracked to the ablation processes induced by the illumination which modified the shape of PA, resulting first in the formation of sharp corners within the gap of the bowtie (this effect is referred to as self-optimization and is believed to be responsible for the signal enhancement) followed by widening the bowtie gap (considered as the degradation causing the decay in THG). The authors claim that *the field enhancement factor gradually decreases with the increase of the curvature radius.* We believe that PLRE contributes to the observed modification of the THG signal, although it acts simultaneously with other effects, in particular the modification of the bowtie gap. Further, the THG signal is contributed by the whole PA cross-section and lacks the spatial resolution required to isolate the PLRE (although for THG the significance of the central hot spot is higher than for the linear response).

Yang et al. [3] studied theoretically the relation between geometric parameters of the bowtie PA and the Purcell factor for enhancement of the dipole radiation. Their study includes three values of the local curvature of the corners at the gap, for which they observed that the Purcell factor increases as the curvature increases. The other parameters influence the exact Purcell-factor–curvature relation (flare angle being a prominent example, see Fig. 5 in Ref. [3]); the PLRE is thus observed but not isolated from other effects. Similar findings are reported by Rogobete et al. [4].

(ii) *Visualization of enhanced field near sharp features of plasmonic antennas.* Such studies are rather frequent, and we discuss in more depth only some influential works.

Schmidt et al. [5] in their instructive paper entitled *Morphing a Plasmonic Nanodisk into a Nanotriangle* studied experimentally by STEM-EELS and theoretically by electrodynamic simulations in total seven silver planar PAs, whose shape was gradually modified from a disk to a triangle (more accurately, a thin triangular prism). The height and the circumference of all PAs were kept constant; it was found that also the energy of the lowest localized surface plasmon (LSP) resonance was nearly identical for all PAs. As the disk is gradually morphed into the triangle, sharp corners appear with locally enhanced loss probability evidenced by both EEL spectra and maps. The spectra show approximately 2.5–3-times enhanced loss probability for the electron beam impinging the corner of the triangle as compared to the disk. This is a nice and convincing visualization of the PLRE. Unfortunately, neither the electric field nor the radius of curvature of the triangle's corner is provided, and the PLRE cannot be thus quantified.

An insightful theoretical demonstration of PLRE on

* krapek@vutbr.cz
† michal.horak2@ceitec.vutbr.cz

the atomistic level is reported by Urbieta et al. [6]. The authors show using time-domain density functional theory the field enhancement at the edges of sodium icosahedral cluster, and interpret it as a non-resonant manifestation of the atomic-scale lightning-rod effect at the facet edges with the effective radius of curvature corresponding to Wigner–Seitz radius of Na atoms (2.08 Å).

Our overview demonstrates that PLRE is frequently discussed and exploited in the field of plasmonics. At the same time, we can see that the understanding of PLRE is restricted to its partial aspects and experimental expression. A rigorous quantitative relation between the local electric field and the local curvature, fully isolated from the concurrent effects influencing the field, is not available. In the present study, we aim to fill this knowledge gap.

S2. LOSS PROBABILITY SPECTRUM IN EXTENDED ENERGY RANGE

Figure S1 shows the loss probability spectra of plasmonic beetroots in the extended energy range. The longitudinal dipole (LD) and longitudinal quadrupole (LQ) resonances are clearly visible. The LD resonance is well separated from the higher modes.

S3. PARAMETERS OF THE PLASMON RESONANCES

Figure S2 shows the parameters of the longitudinal dipole (LD) LSP resonance obtained from fitting the peak in the loss probability around 1.1 eV (both experimental and calculated) with Lorentzian profiles. The parameters are obtained for two series of plasmonic beetroots (PBs) with $r_{\mathrm{fix}} = 70$ nm or 50 nm and r_{var} varied from 20 nm to r_{fix}, and the electron beam positioned near either of the terminations of the PB, as indicated in the inset of Fig. S2(c). The central energies and Q factors [defined as the central energy divided by the full width at half maximum (FWHM)] are shown in Fig. S2(a) and S2(b). Both quantities are rather independent of both r_{fix} and r_{var}, suggesting that the LD LSP resonances in all PBs are as similar as possible, and any variations of their properties (in particular the electric field distribution) unrelated to the local curvature shall be suppressed as much as possible. Both quantities are also independent of the position of the electron beam, demonstrating that the same LD LSP resonance is excited for both electron beam positions, which is also an important factor ensuring the comparability of the electric fields corresponding to both positions. For the central energies, we observe a good agreement between the simulations and the experiment. The Q factors differ due to instrumental broadening relevant to the experiment. This issue is in more detail discussed in Fig. S3 and the related discussion.

Figure S2(c,d) shows the peak amplitude of the loss probability for the two positions of the electron beam. When r_{var} is increased, the loss probability for the electron beam near the respective termination decreases (a consequence of PLRE), while the loss probability for the electron beam at the opposite termination (the reference termination with the fixed radius r_{fix}) is nearly constant, demonstrating that the additional effects influencing the electric near field beyond the local geometry are efficiently excluded in the PB. Since the relation between the loss probability and the induced electric near field is rather complex [7, 8], Fig. S2(c,d) can be viewed as an indirect manifestation of the PLRE, but for the quantitative description the analysis of the electric field is inevitable. The differences between calculated and experimental values are again related to the instrumental broadening.

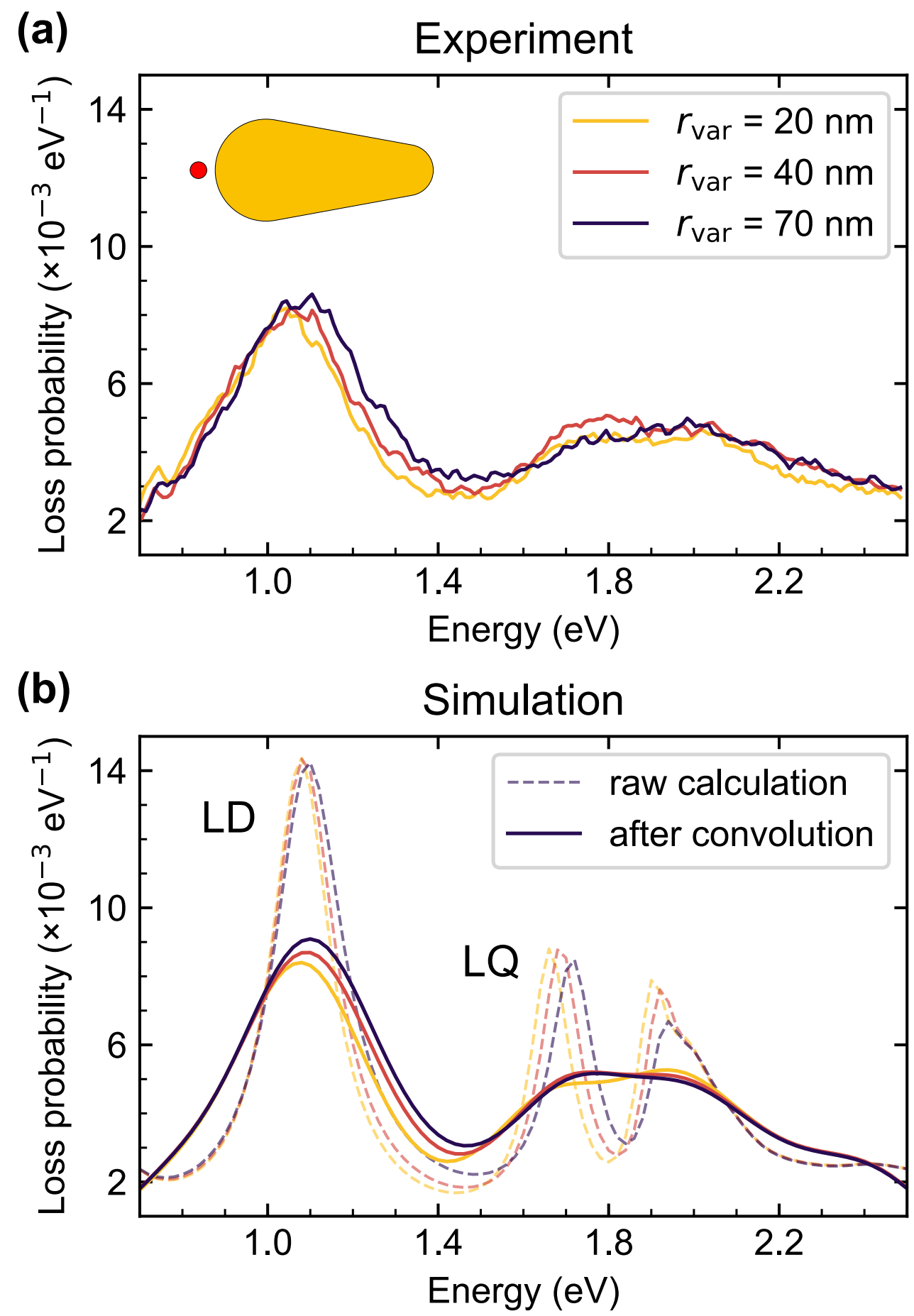

FIG. S1. Loss probability spectra for PBs with $r_{\mathrm{fix}} = 70$ nm and the electron beam position indicated in the inset (10 nm from the PB boundary). (a) Experimental spectra for three values of r_{var}. (b) Calculated spectra (solid lines) and calculated spectra convolved with a Gaussian (full width at half maximum of 0.27 eV) representing instrumental broadening.

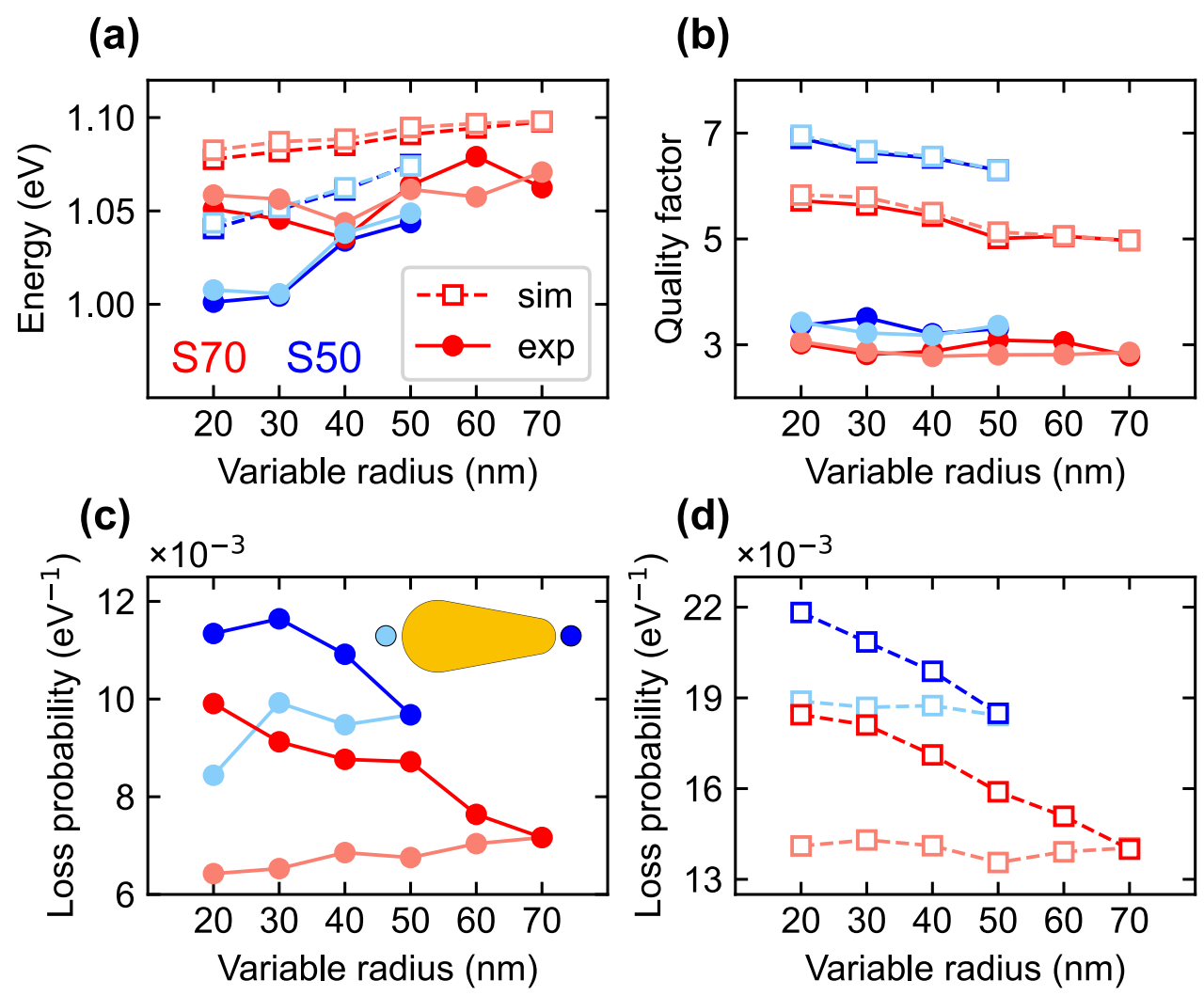

FIG. S2. Parameters of the LD LSP resonance: (a) central energy, (b) Q factor, (c,d) peak loss probability [experimental (c) and calculated (d)]. In all panels, full circles represent experimental values and empty squares represent theoretical values. The red and blue color corresponds to the PB series with $r_{\rm fix}$ of 70 nm and 50 nm, respectively. Light and dark colors refer to the electron beam position near the left (with the radius $r_{\rm fix}$) and right ($r_{\rm var}$) termination of the PB as indicated in the inset of the panel (c).

S4. EXPERIMENTAL VERIFICATION OF SIMULATIONS

Electrodynamic simulations predict the linear optical response of PAs rather reliably as long as the quantum effects are negligible and realistic shapes, dimensions, and dielectric functions are utilized. Nevertheless, it is worth verifying the simulations and assessing their accuracy for each specific study. To this end, we compare the loss probability obtained from simulations and retrieved from an experiment.

Fig. S3(a) compares the spectral dependence of the loss probability obtained for the electron beam located 10 nm from the edge of the plasmonic beetroot at the position indicated in the inset of Fig. S3(a). To account for the experimental spectral broadening, the loss probability obtained from simulations is convolved with a Gaussian with the FWHM of 0.27 eV. The convolved spectrum (green line) agrees very well with the experimental spectrum (red line) in terms of the position and width of spectral features, and reasonably well in their intensity. We note that to facilitate the comparison, a constant value of 1.5×10^{-3} eV^{-1} was added to the experimental spectrum, so the agreement of the intensities is slightly worse than it looks in the graphical comparison. The choice of the Gaussian is justified by a good agreement between the experimental and convolved data. The instrumental function itself is a convolution of several contributions, among which the monochromator and the spectrometer contributions dominate. The spectral profile of the experimental data is further influenced by the data processing (zero-loss peak removal and background subtraction). The overall complexity of the spectral broadening prevents us from using a rigorous model.

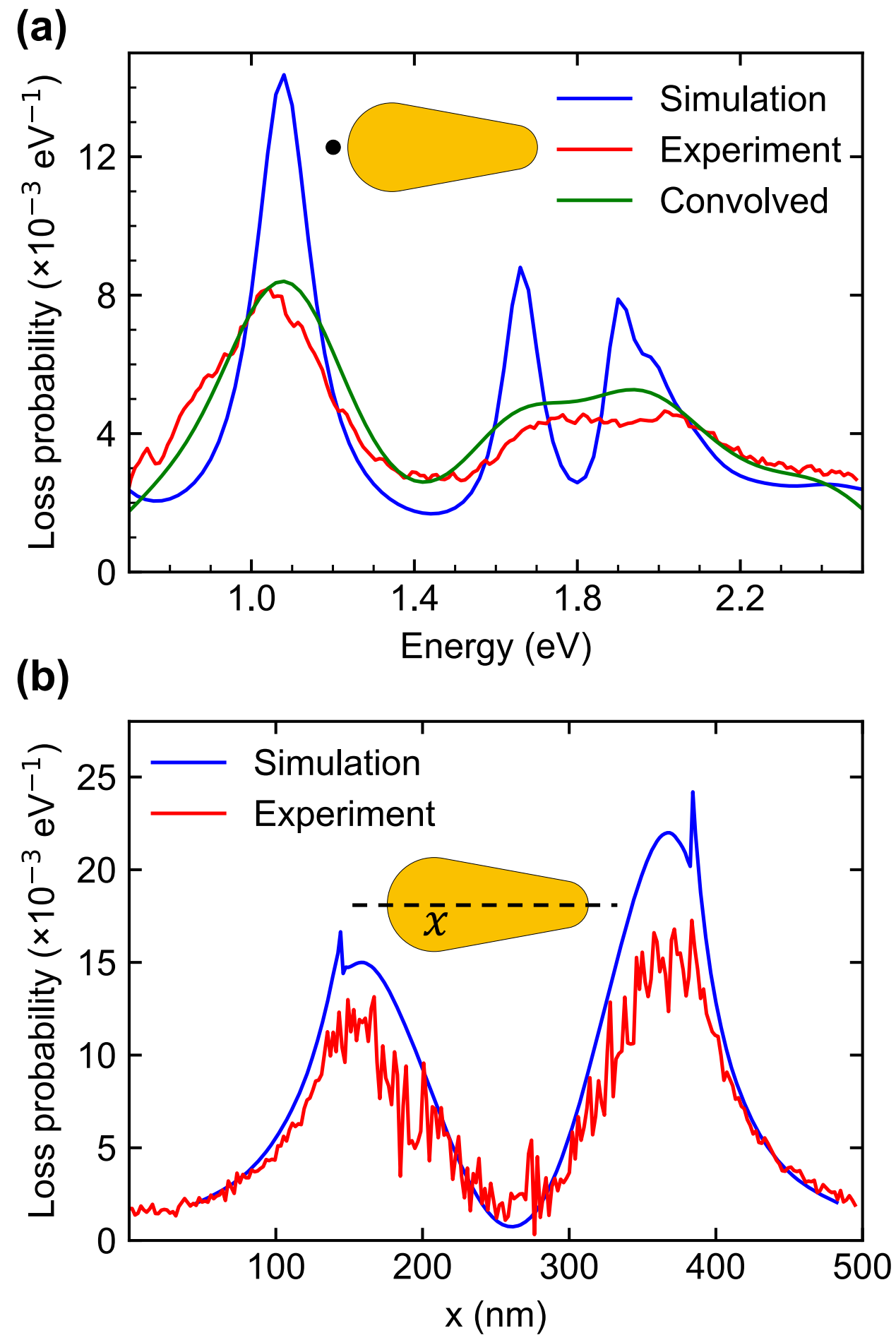

FIG. S3. Comparison of theoretical and experimental loss probabilities. (a) EEL spectrum of a plasmonic beetroot ($r_{\rm fix} = 70$ nm, $r_{\rm var} = 20$ nm) for the electron beam position indicated in the inset: the theoretical spectrum (blue line) is convolved with a Gaussian (FWHM of 0.27 eV) representing the instrumental broadening (green line) and compared to the experimental spectrum (red line). (b) Linear cross-section of the theoretical (blue line) and experimental (red line) loss probability along the axis of the same plasmonic beetroot (shown in the inset) at the energy of the longitudinal dipole mode (around 1.1 eV).

Fig. S3(b) compares the spatial dependence of the loss probability along the axis of the plasmonic beetroot (as indicated in the respective inset) at the energy of the longitudinal dipole mode. Both theoretical and experimental profiles are comparable, with two maxima at the opposite sides of the beetroot characteristic for the longitudinal dipole mode. We note that in the case of the spatial dependence we have not applied the subtraction of background to the experimental data and the convolution

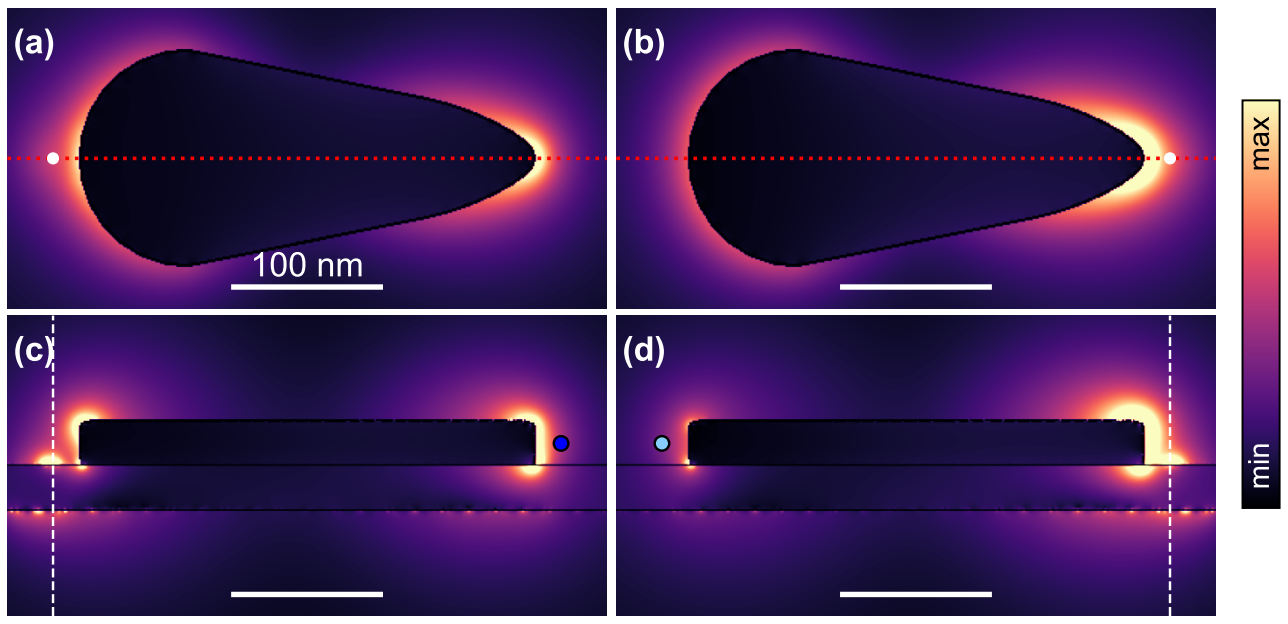

FIG. S4. The magnitude of the electric field induced by the electron beam in the plasmonic beetroot ($r_{\rm fix} = 70$ nm, $r_{\rm var} = 20$ nm; the excitation field of the electron beam is excluded) at the energy of the longitudinal dipole mode (around 1.1 eV). The electron beam is positioned at the left side of the PB with the radius $r_{\rm fix}$ [left panels (a,c)] or at the right side of the PB with the radius $r_{\rm var}$ [right panels (b,d)], always at the distance of 20 nm from the PB boundary. The position of the electron beam is indicated by white spots and white dashed lines. Top panels (a,b) show the top view at the central height of the PB with the boundary of the PB indicated by a black line and the central longitudinal axis of the PB indicated by a red dashed line. Bottom panels (c,d) show the side view in the central plane of the PB. Black lines indicate the boundaries of the PB and the substrate. Blue spots indicate the position where the field shown in Fig. 4 is evaluated.

to the theoretical data; consequently, this comparison is not fully quantitative.

Very good agreement between theoretical and experimental loss probabilities in both spectral and spatial domains verifies the correctness and accuracy of the simulations, as well as their predictive power for full vectorial characterization of the electromagnetic near-field, which is not directly accessible with our experimental setup.

S5. ELECTRIC FIELD

Figure S4 shows the magnitude of the electric field induced by the electron beam in and around the PB ($r_{\rm fix} = 70$ nm, $r_{\rm var} = 20$ nm) at the energy of the longitudinal dipole mode (around 1.1 eV). The field of the electron beam (i.e., the field produced by the same electron beam in a vacuum) is excluded from the displayed field (i.e., it is subtracted from the total calculated field). The beam is positioned either near the left part of the PB with the radius $r_{\rm fix} = 70$ nm (panels a,c) or near the right part of the PB with the radius $r_{\rm var} = 20$ nm (panels b,d). Detailed inspection of the field allows us to formulate the following statements: (i) The field distribution with two maxima at opposite sides of PB verifies the longitudinal dipole character of the LSP resonance at the considered energy. (ii) We observe a clear asymmetry of the field between the two terminations. The field at $r_{\rm var} = 20$ nm is stronger than the field at $r_{\rm fix} = 70$ nm. This is the manifestation of PLRE. (iii) We observe another type of asymmetry related to the position of the electron beam. The induced field is relatively higher at the side of the PB where the electron beam is positioned. We suppose that this fact might be attributed to lossy nature of LSP resonances. There is a practical implication relevant to our study: in the enhancement factor discussed in the main paper, we compare the fields induced at both sides of the PB. It is reasonable to define this factor so that it is equal to one for the symmetric PB (where $r_{\rm var} = r_{\rm fix}$). However, this would not hold if we kept the position of the electron beam. We therefore always position the electron beam to the opposite side to that one where the field is recorded. (iv) We observe a weak but discernible oscillating surface field at the boundary between the substrate and the vacuum. It is probably related to some numerical instability. (v) We observe particularly large fields close to the corners at the bottom and top of the PB (visible in panels b,d) and near the spot where the electron beam crosses the substrate. These fields can suffer from large numerical errors and we exclude them from further analysis. (vi) The field does not penetrate the metal, as evidenced by the dark color of the PB.

S6. EMPIRICAL MODEL FOR ELECTRIC FIELD

As we discuss in the main paper (see Fig. 5 and the related discussion), the induced electric field at the axis of the PB can be described by a simple empirical model

$$E(x) = \frac{Q_{\rm eff}}{(r_{\rm eff} + x)^d}, \tag{S1}$$

where $Q_{\rm eff}$ represents the effective charge induced inside the PB at the distance $r_{\rm eff}$ from the PB boundary (the so-called effective radius), d is the so-called dimensionality parameter, and x is the distance from the PB boundary into the surrounding vacuum. We stress that the empirical model only empirically represents the observations based on full electrodynamic simulations. It cannot be rigorously derived from physical principles, although it was inspired by the physics of the electrostatic lightning-rod effect.

The effective radius $r_{\rm eff}$ represents the position of the effective charge $Q_{\rm eff}$ – specifically, its distance from the boundary of the PB. It is inspired by the electrostatic model of the lightning-rod effect presented in Fig. 1(a) in the main paper. In this model, the real charge, distributed homogeneously over the spherical surface, can be substituted by the effective point charge located at the center of the sphere, i.e., at a distance equal to the radius from the sphere boundary. Analogously, we label the distance of the effective charge from the boundary of the plasmonic antenna as the effective radius also in the case of PLRE.

We have also tested a slightly more complex model that reflects the exponential decay of the field of SPP,

r_{var} (nm)	20	30	40	50	60	70
Q_{eff} (a.u.)	7.4	8.5	8.2	8.0	7.5	6.7
d	1.24	1.26	1.25	1.24	1.23	1.20
r_{eff} (nm)	6.3	8.2	9.8	11.7	13.7	15.8

TABLE S1. Parameter estimators for the model described by Eq. S1 applied to the distance range of 10–150 nm.

r_{var} (nm)	20	30	40	50	60	70
Q_{eff} (a.u.)	5.1	5.9	5.6	5.4	5.4	5.5
d	1.16	1.19	1.18	1.17	1.16	1.17
r_{eff} (nm)	3.2	5.2	6.5	8.1	10.3	13.7

TABLE S2. Parameter estimators for the model described by Eq. S1 applied to the distance range of 20–150 nm.

reading

$$E(x) = \frac{Q_{\text{eff}}}{(r_{\text{eff}} + x)^d} \times \exp(-\kappa x), \tag{S2}$$

where κ is the decay parameter of SPP. The value of κ can be calculated for the planar metal-dielectric interface (see the main paper for details) and it takes the value of 9.5×10^{-4} nm^{-1} (with the imaginary part neglected) for our value of the dielectric function of gold $\varepsilon_m = -33.6 + 4.8\text{i}$ at the photon energy of 1.08 eV. However, in our model, we set κ as a free parameter with the value to be fit to the calculated spatial profile of the field $E(x)$. This is reflecting the finite curvature of the interface and the proximity of the substrate, both of which might alter the value of κ valid for the planar interface.

It is worth considering the units of the model parameters. We use arbitrary units for the field E. Therefore, the charge Q_{eff} is also expressed in arbitrary units. The dimensionality parameter d is dimensionless. The distance x is expressed in nm, so is the effective radius r_{eff} and κ is expressed in nm^{-1}.

We have applied both models to the calculated spatial field profile $E(x)$ with x either in the range 10–150 nm or 20–150 nm, the PB with $r_{\text{fix}} = 70$ nm and $r_{\text{var}} = 20$ nm, and the energy of the longitudinal dipole mode (around 1.1 eV). The values of the parameters obtained by least-square fitting are summarized in Tables S1–S4.

Both models fit the $E(x)$ dependence very well in both distance ranges. However, the latter model (defined by Eq. S2) seems to contain too many parameters with strong correlations between their estimators. In the distance between 10–20 nm, the field seems to somewhat

r_{var} (nm)	20	30	40	50	60	70
Q_{eff} (a.u.)	20	24	26	28	25	15
d	1.50	1.52	1.54	1.55	1.51	1.39
r_{eff} (nm)	10.0	12.3	14.5	17.2	19.2	19.8
κ ($10^{-3} \cdot$ nm^{-1})	−2.6	−2.5	−2.6	−2.6	−2.3	−1.5

TABLE S3. Parameter estimators for the model described by Eq. S2 applied to the distance range of 10–150 nm.

r_{var} (nm)	20	30	40	50	60	70
Q_{eff} (a.u.)	4.1	4.3	4.0	4.2	3.8	2.9
d	1.11	1.11	1.10	1.11	1.08	1.01
r_{eff} (nm)	2.0	3.4	4.6	6.5	8.0	9.1
κ ($10^{-3} \cdot$ nm^{-1})	0.4	0.6	0.6	0.4	0.6	1.0

TABLE S4. Parameter estimators for the model described by Eq. S2 applied to the distance range of 20–150 nm.

deviate from the single charge. This deviation mildly affects the estimators of the parameters in the model of Eq. S1 (as evidenced by mild differences between the estimators in Tables S1 and S2), but there is a significant effect on the estimators of the parameters in the model of Eq. S2 (evidenced by significant differences between the values of the estimators in Tables S3 and S4, in particular the negative values of κ for the distance range of 10–150 nm). We therefore prioritize the more simple model of Eq. S1.

The errors of parameter estimators are dominated by the systematic error of the model, which can be inferred from the comparison of the estimators obtained for different distance ranges. For example, the effective radius r_{eff} obtained for $r_{\text{var}} = 70$ nm varies between 13.7 nm and 15.8 nm for the model of Eq. S1 and between 9.1 nm and 19.8 nm for the model of Eq. S2 (the larger spread obtained for the latter model illustrates our suggestion that the model is overparametrized). The largest difference for the model of Eq. S1 is observed for the $r_{\text{var}} = 20$ nm, where r_{eff} varies between 3.2 nm and 6.3 nm. The spread of the values of 3.1 nm represents 15 % of the corresponding geometric radius $r_{\text{var}} = 20$ nm, which we consider acceptable. The stochastic errors of the parameter estimators are negligible, and we do not show them. In all cases, the effective radius r_{eff} uniformly scales with geometric radius r_{var} and is significantly smaller, although the exact values vary. This supports the robustness of our conclusions related to the existence of the effective radius and its relation to the geometric radius of curvature (i.e., the effective radius is smaller than the geometric radius) presented in the main paper. We note that in the main paper, we utilize the values displayed in Table S1.

It is important to emphasize that the model is empirical, rather than fully physical, although it is inspired by physical principles. We highlight the following key limitations of the model: (1) The model employs an electrostatic framework, where charge serves as the source of the electric field, to describe the non-stationary case with an intertwined electromagnetic field. (2) We represent a realistic distribution of the induced charge by a single charge (with the empirical dimensionality parameter increasing somewhat the flexibility of the model and allowing to smoothly vary the nature of the effective charge between a point charge and a linear charge).

While the model is validated by good agreement with the electric field obtained from full electromagnetic simulations, its parameters should be interpreted with care

as empirical, rather than as real physical quantities.

S7. UNIVERSALITY OF PLASMONIC LIGHTNING-ROD EFFECT

In the manuscript and the previous sections of the Supplemental Material, we addressed PLRE thoroughly but only for a limited set of PAs. Here we aim to show that the effect is universal and applicable to arbitrary PAs. We assess the role of the substrate and show the independence of PLRE of a specific excitation field. To gain a better insight into the similarities and differences between PLRE and electrostatic lightning-rod effect (ELRE), we investigate plasmonic beetroots and plasmonic dumbbells with varied length.

In this section, we exploit a simplified methodology comprising electromagnetic simulations, while experimental verification is not performed. Further, to simplify the simulations, the substrate (a TEM membrane) was not included in the PAs with a varied length (discussed in subsections S7 C and S7 D).

A. Substrate

PAs are very sensitive to their surrounding environment, including the substrate. Our choice of the substrate – a silicon-nitride TEM membrane with a thickness of 30 nm – was determined by the need to inspect the fabricated samples by STEM-EELS. To briefly assess the role of the substrate in PLRE, we compare the properties of PBs with this TEM substrate and without any substrate ($r_{\mathrm{fix}} = 50$ nm, r_{var} varied between 50 nm and 20 nm). We utilized the same procedure as in the main paper: From calculated EEL spectra we have determined the central energy of the longitudinal dipole resonance, for that energy we calculated the distribution of the induced electric field, and finally, we evaluated enhancement factors and parameters of the empirical model of the field. The results are summarized in Fig. S5 and Table S5.

The induced electric field with and without the substrate is qualitatively similar with minor quantitative differences (e.g., 5 % difference in the magnitude is observed for $r_{\mathrm{var}} = 20$ nm at the distance $x = 10$ nm. To facilitate the comparison of the field spatial dependences, we have renormalized both fields to the same amplitude at a distance of $x = 10$ nm from the PB, as shown in Fig. S5(a). The differences between the renormalized fields are hardly discernible; a closer inspection reveals relative differences between the fields below 5 % for the distance range up to 100 nm.

Similarly, the enhancement factors [Fig. S5(b)] show similar qualitative trends with minor quantitative differences, e.g., the values of 1.43 and 1.35 are observed for the case with and without the substrate, respectively, for $r_{\mathrm{var}} = 20$ nm. The differences in the parameter estimators (Tab. S5) are more pronounced. Without the substrate, the effective radii r_{eff} are considerably larger (up to 100 %, which is still significantly lower than the geometric radii), and the dimensionality factors d are also larger (by about 25 %). This fact illustrates that our model is somewhat overparameterized, with a strong correlation between r_{eff} and d, which are thus rather susceptible to minor variations of the field.

We note that the removal of the substrate resulted, among other changes, in the blue shift of the longitudinal dipole mode by about 0.15 eV. This change in the resonance energy is accompanied by the change in the dielectric function of gold (towards a "worse" metal with a less negative real part). Consequently, there is an in-

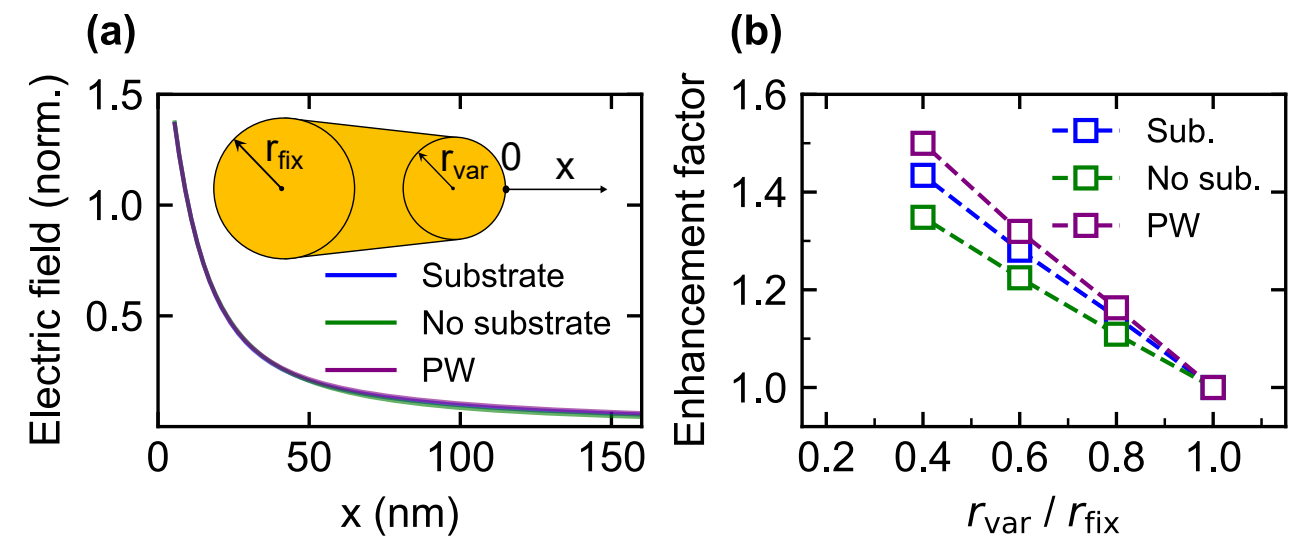

FIG. S5. The role of the substrate and the excitation: a comparison of PBs ($r_{\mathrm{fix}} = 50$ nm, r_{var} varied between 50 nm and 20 nm) with the TEM substrate (a silicon-nitride TEM membrane with a thickness of 30 nm) excited by the electron beam, and without any substrate, excited either by the electron beam or by a plane wave. (a) The induced electric field near the variable-radius termination analogous to Fig. 5 of the main paper for $r_{\mathrm{var}} = 20$ nm: TEM substrate (blue line); no substrate, electron beam (green line); no substrate, plane wave (violet line). All fields are normalized to unity at the distance from the variable-radius termination $x = 10$ nm. (b) Enhancement factors analogous to Fig. 4(d) of the main paper: TEM substrate [blue, identical to Fig. 4(d)]; no substrate, electron beam (green); no substrate, plane wave (violet).

r_{var} (nm)	20	30	40	50
d^{S}	1.28	1.27	1.28	1.25
$d^{\mathrm{S,PW}}$	1.19	1.18	1.18	1.16
d^{NS}	1.53	1.58	1.61	1.64
$d^{\mathrm{NS,PW}}$	1.56	1.60	1.64	1.68
$r_{\mathrm{eff}}^{\mathrm{S}}$ (nm)	6.5	8.5	11.2	13.9
$r_{\mathrm{eff}}^{\mathrm{S,PW}}$ (nm)	5.1	6.9	9.3	11.8
$r_{\mathrm{eff}}^{\mathrm{NS}}$ (nm)	12.2	15.7	19.7	24.8
$r_{\mathrm{eff}}^{\mathrm{NS,PW}}$ (nm)	12.4.	16.0	20.1	25.3

TABLE S5. Selected parameter estimators for the model described by Eq. S1 applied to the distance range of 10–150 nm for the fields shown in Fig. S5(a). The superscripts S and NS correspond to the case with the substrate and with no substrate, respectively. The superscript PW corresponds to the plane wave excitation (otherwise, an electron beam near the fixed-radius termination is used).

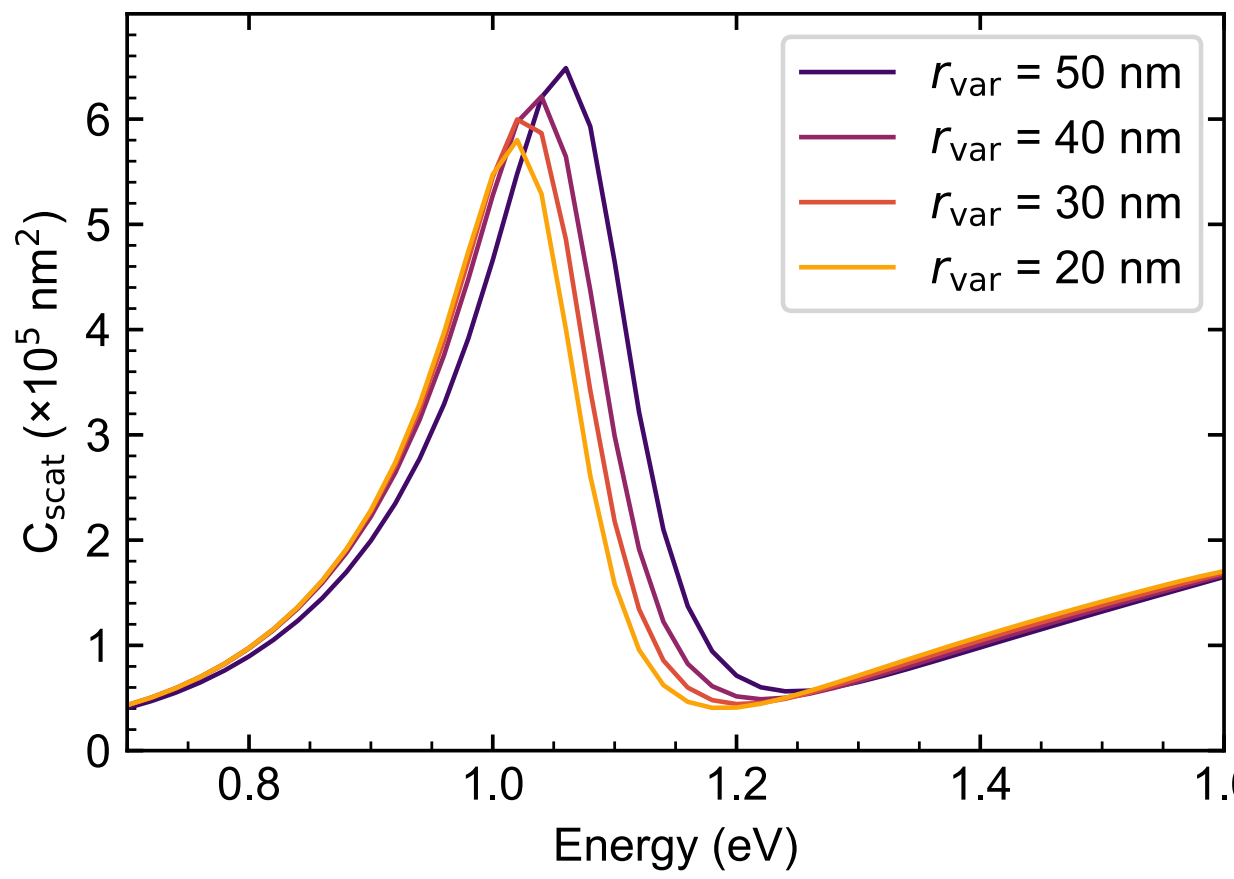

FIG. S6. The calculated scattering cross section of the plasmonic beetroot ($r_{\rm fix}$ = 50 nm, $r_{\rm var}$ varied from 50 nm to 20 nm.

direct effect on the induced electric field in addition to the direct effect related to the modified dielectric environment, and the observed role of the substrate has to be interpreted cautiously.

B. Plane wave excitation

The excitation of the plasmonic antenna (PA) with an electron beam (EB) is relevant for our experimental approach. In numerous applications, PAs interact with an electromagnetic plane wave (PW), including plasmon-enhanced reflection or absorption spectroscopy, Raman spectroscopy, or photoluminescence spectroscopy (more specifically, its absorption process). Furthermore, the PW is a delocalized excitation that interacts indiscriminately with the entire area of a PA, resolving the issue with the EB positioned to the same or the opposite termination as that where the field is evaluated. It is therefore worth comparing the electric fields induced in PBs by the EB and by an electromagnetic PW. We consider the polarization parallel to the long axis of the PB. The results are summarized in Fig. S5 and Table S5.

The induced electric field is significantly larger for the PW excitation, which is in our setup significantly stronger than the EB excitation. When the fields are renormalized to the same amplitude at the distance of 10 nm from the PB [as shown in Fig. S5(b)], we observe minor differences in their spatial dependences below 2 % (i.e., the difference is significantly smaller than the difference between the present and missing substrate discussed above). The parameter estimators $r_{\rm eff}$ and d are nearly identical for both excitations, underlining the generality of the involved empirical model of the induced electric field.

For completeness, we also show the calculated scattering cross section in Fig. S7 and the field distribution in

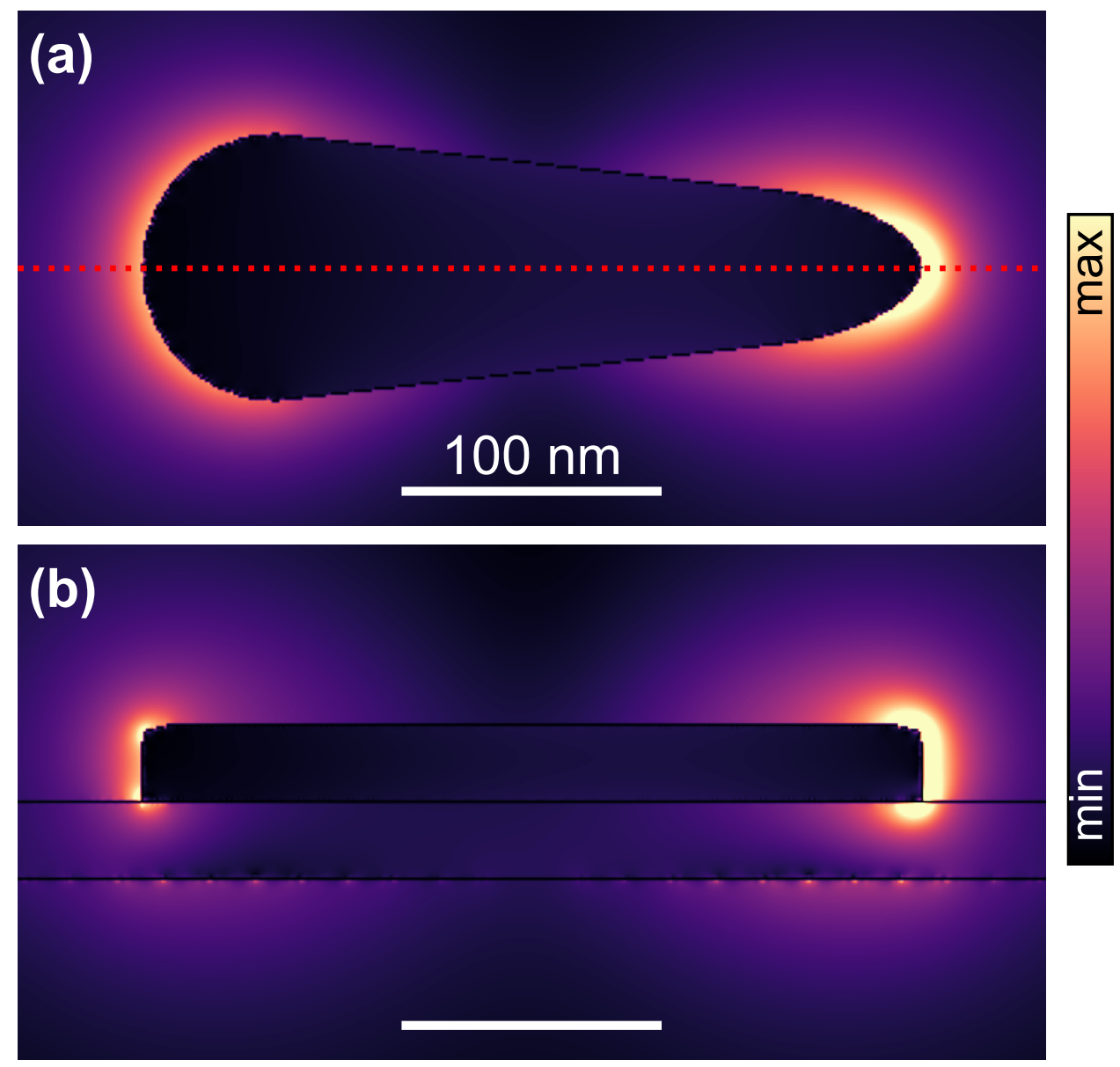

FIG. S7. The magnitude of the electric field induced by the plane wave in the plasmonic beetroot ($r_{\rm fix}$ = 50 nm, $r_{\rm var}$ = 20 nm; the excitation field is excluded) at the energy of the longitudinal dipole mode (1.04 eV). The plane wave is polarized parallel to the long axis of the PB. (a) The top view at the central height of the PB, with the boundary of the PB indicated by a black line and the central longitudinal axis of the PB indicated by a red dashed line. (b) The side view in the central plane of the PB. Black lines indicate the boundaries of the PB and the substrate.

Fig. S6.

C. Length

When comparing ELRE and PLRE, we identified a principal difference in the position of the effective induced charge. In our analysis of PLRE, the effective charge was located near the surface of PBs (thus reducing their effective radius). On the other hand, in ELRE represented by the case of two independent metallic spheres [see Fig. 1(a) in the main paper], the effective charge was located in the centers of the spheres. However, the real charge is also located at the surface of the spheres. It is the specific geometry that allows us to represent a homogeneous surface charge on the spheres' surfaces by the effective charge in their centers. Therefore, we would like to investigate whether, by a proper modification of the geometry, the central position of the effective charge can also be achieved in PLRE.

One of the reviewers of our paper proposed a hypothesis that the near-surface location of the effective charge in PLRE can be related to a low distance between two terminations, whose fields are therefore not independent (in contrast to the case of ELRE where the spheres are connected with a *long* wire). This hypothesis suggests that

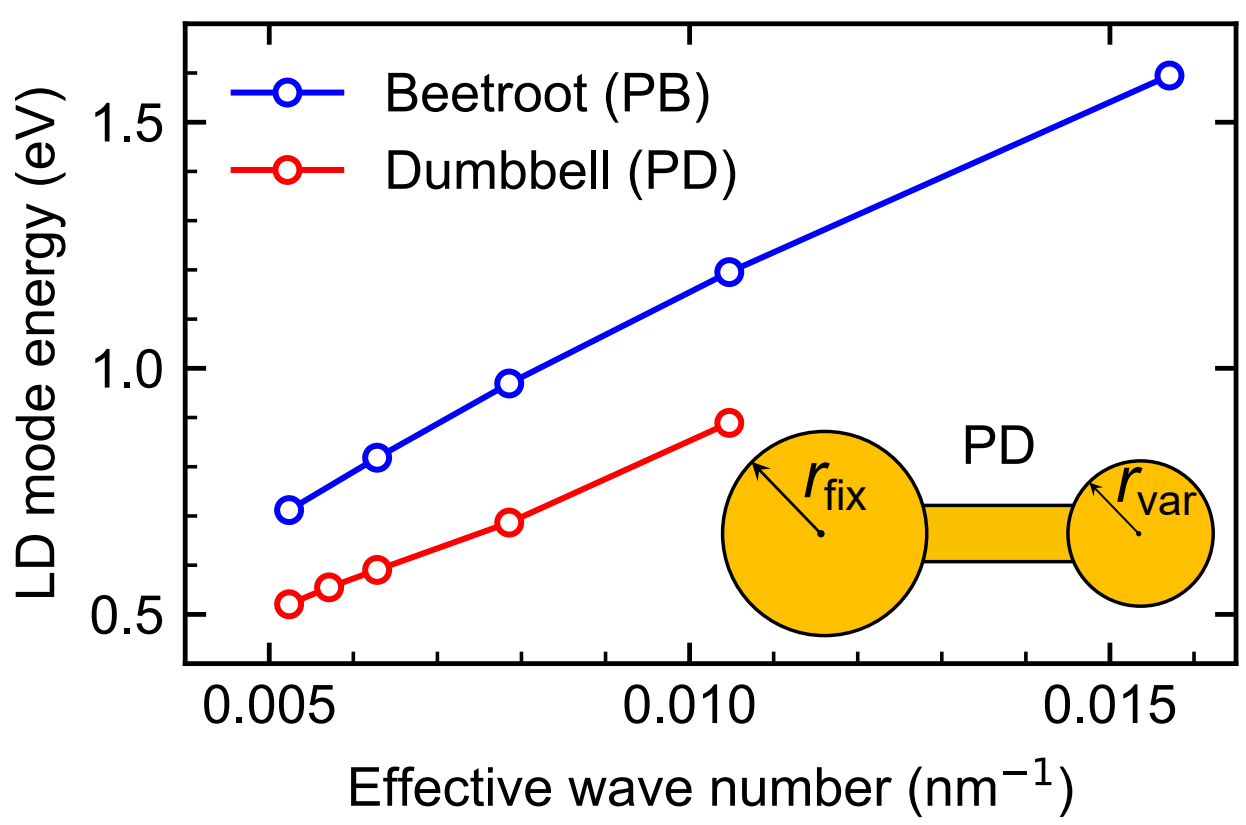

FIG. S8. Effective dispersion relation of the LD mode in PB (blue line) and plasmonic dumbbell (PD) (red line): The energy of the mode as a function of effective wave number $k = 2\pi/\lambda$ considering that the length of the PA L equals half of the effective wavelength λ, i.e., $L = \lambda/2$. The concept of the effective dispersion relation was established in Ref. [9]. The inset schematically shows the PD shape.

L (nm)	150	200	300	400	500	600
d	1.69	1.63	1.53	1.49	1.46	1.44
r_{eff} (nm)	12.3	12.8	12.2	11.9	11.3	11.0
EF	1.37	1.35	1.35	1.36	1.37	1.37

TABLE S6. Selected parameter estimators for the model described by Eq. S1 applied to the distance range of 10–150 nm and the enhancement factor EF at the distance of 10 nm from the PB boundary for the fields of the LD mode supported by PBs ($r_{\mathrm{fix}} = 50$ nm, $r_{\mathrm{var}} = 20$ nm) with the length L varied from 150 nm to 600 nm. The electron beam near the fixed-radius termination was used as the excitation, and the field was evaluated near the variable-radius termination.

when the PB is made longer (and the mutual interaction of induced charges in both terminations is weakened), the charges would be shifted towards the central position reflected in the increase of the effective radii (r_{eff}) towards the geometric radii (r_{fix} or r_{var}). On the other hand, the charges on the opposite terminations of the PB for a dipole mode in PLRE have opposite signs, and their mutual interaction is attractive (in contrast to ELRE, where both charges have the same sign and their interaction is repulsive), and weakening of the interaction between them can enhance the separation of the charges, reflected in further decrease of the effective radii r_{eff}.

To analyze the above-mentioned hypotheses, we gradually modified the length of a PB ($r_{\mathrm{fix}} = 50$ nm, $r_{\mathrm{var}} = 20$ nm) from 150 nm to 600 nm (with the length of 300 nm corresponding, except for the absence of substrate, to PBs discussed in the main paper). The modification of the length results in the shift of the resonance energy of the LD mode, as shown in Fig. S8. We have evaluated the effective radii at the r_{var} termination and the enhancement factors. The results are summarized in Table S6.

L (nm)	300	400	500	550	600	650
d	1.85	1.81	1.77	1.75	1.74	1.73
r_{eff} (nm)	26.6	26.1	25.3	24.9	24.6	24.5
EF	1.31	1.36	1.34	1.34	1.34	1.34

TABLE S7. Selected parameter estimators for the model described by Eq. S1 applied to the distance range of 10–150 nm and the enhancement factor EF at the distance of 10 nm from the PD boundary for the fields of the LD mode supported by PDs ($r_{\mathrm{fix}} = 70$ nm, $r_{\mathrm{var}} = 40$ nm) with the length L varied from 300 nm to 650 nm. The electron beam near the fixed-radius termination was used as the excitation, and the field was evaluated near the variable-radius termination.

The effective radius is nearly independent of the length of the PB. Minor variations of the effective radius (below 10 % of its mean value) seem to positively correlate with the variations in the dimensionality parameters (as in the case of Fig. S5), further obscuring a possible tiny effect of the length. Similarly to the effective radius, the enhancement factor is also rather independent of the PB length.

D. Dumbbells

Another modification of a PB towards the ELRE case of two independent spheres consists in thinning the part connecting the two cylindrical terminations. In this way, a so-called plasmonic dumbbell (PD) is formed, schematically shown in the inset of Fig. S8. In this subsection, we focus on a PD with the radii $r_{\mathrm{fix}} = 70$ nm and $r_{\mathrm{var}} = 40$ nm and the width of the connecting part of 20 nm. The length of the PD was varied between 300 nm and 650 nm. The LD mode energies are plotted in Fig. S8 and the values of the effective radius and the enhancement factor are summarized in Table S7. Similar to the case of PB, both the effective radius and the enhancement factor are nearly independent of the length of the PD.

Further, it is instructive to compare PBs and PDs. For all the PAs considered in Secs. S7 C and S7 D (and related Tables S6 and S7), the approximate relation between the effective radius r_{eff} and the geometric radius r_{var} reads $r_{\mathrm{eff}} \approx 0.6 r_{\mathrm{var}}$, suggesting that there is no systematic difference between r_{eff} values of PBs and PDs.

The observations of Secs. S7 C and S7 D allow us to draw two conclusions: (i) The effective radius is a robust concept, closely related to the local geometric curvature of the PB but mostly independent of its length (and, consequently, the energy of the LD mode). (ii) The difference in the distribution of the induced charge between ELRE and PLRE is not a purely geometric effect. When changing the PB geometry utilized in PLRE towards two independent spheres utilized in ELRE (either by elongating or thinning the central part of the PB connecting the cylindrical terminations), no systematic convergence of the effective radius to the geometric radius is observed.

We suppose that the difference originates from the contrast between the dynamic nature of PLRE with oscillating charge and static ELRE, where the charge reaches the equilibrium position.

E. Angular dependence

All our previous discussions targeted the field at the long axis of PAs (either PBs or PDs). Here, we investigate the possibility of extending our description of PLRE to the full horizontal plane of PAs. We utilize cylindrical coordinate systems associated with the centers of cylindrical termination. We calculate the test field and the reference field at certain points with the same cylindrical coordinates at the variable-radius termination (the test field) and the fixed-radius termination (the reference field). The results displayed in Fig. S9 for a PB ($r_{\text{fix}} = 50$ nm, $r_{\text{var}} = 20$ nm, $L = 300$ nm, plane-wave excitation, no substrate, the energy of the LD mode) indicate a weak angular dependence of both the test field and the reference field. The corresponding enhancement factor EF (the ratio of the test field and the reference field) also exhibits a weak angular dependence (e.g., for the distance 10 nm from the PB boundary it varies between 1.57 at the zero angle and 1.65 at the angle of 90°), suggesting that PLRE is rather an isotropic effect.

We note that since we focus on planar PAs without well-defined vertical curvature (a zero vertical curvature of the faces is followed by abrupt edges of high curvature), we cannot address PLRE in full three-dimensional space.

[1] A. García-Etxarri, P. Apell, M. Käll, and J. Aizpurua, Opt. Express **20**, 25201 (2012).

[2] L. Shi, B. Iwan, R. Nicolas, Q. Ripault, J. R. C. Andrade, S. Han, H. Kim, W. Boutu, D. Franz, T. Heidenblut, C. Reinhardt, B. Bastiaens, T. Nagy, I. Babushkin, U. Morgner, S.-W. Kim, G. Steinmeyer, H. Merdji, and M. Kovacev, Optica **4**, 1038 (2017).

[3] J. Yang, F. Kong, K. Li, and J. Zhao, Prog. Electromagn. Res. Lett. **44**, 93 (2014).

[4] L. Rogobete, F. Kaminski, M. Agio, and V. Sandoghdar, Opt. Lett. **32**, 1623 (2007).

[5] F. P. Schmidt, H. Ditlbacher, F. Hofer, J. R. Krenn, and U. Hohenester, Nano Lett. **14**, 4810 (2014).

[6] M. Urbieta, M. Barbry, Y. Zhang, P. Koval, D. Sánchez-Portal, N. Zabala, and J. Aizpurua, ACS Nano **12**, 585 (2018).

[7] F. J. García de Abajo, Rev. Mod. Phys. **82**, 209 (2010).

[8] V. Křápek, A. Konečná, M. Horák, F. Ligmajer, M. Stöger-Pollach, M. Hrtoň, J. Babocký, and T. Šikola, Nanophotonics **9**, 623 (2020).

[9] F.-P. Schmidt, H. Ditlbacher, U. Hohenester, A. Hohenau, F. Hofer, and J. R. Krenn, Nature Communications **5**, 3604 (2014).

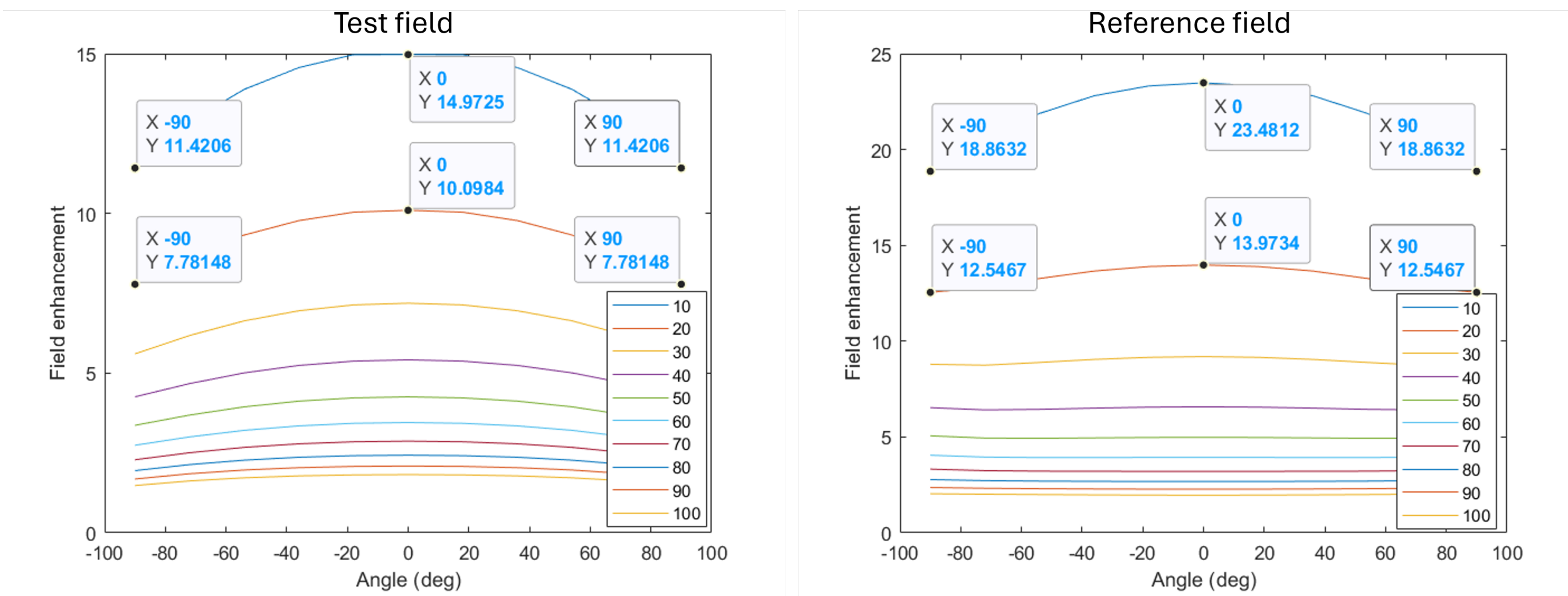

FIG. S9. Angular dependence of the test field and the reference field in a PB ($r_{\rm fix} = 50$ nm, $r_{\rm var} = 20$ nm, $L = 300$ nm, no substrate) excited by a plane wave and taken at the energy of the LD mode. Cylindrical coordinates associated with the centers of the variable-radius termination for the test field and the fixed-radius termination for the reference field are used. Zero angle corresponds to the long axis of the PB. All electric fields are normalized by the electric field of the incident plane wave, and are thus labeled as field enhancements. Different colors correspond to distances from the PB boundary, ranging from 10 nm (light blue) to 100 nm (yellow), as indicated in the boxes.
